# The building blocks of caveolae revealed: caveolins finally take center stage

**DOI:** 10.1042/BST20221298

**Published:** 2023-04-21

**Authors:** Anne K. Kenworthy

**Affiliations:** 1 Center for Membrane and Cell Physiology, University of Virginia, Charlottesville, VA, U.S.A.; 2 Department of Molecular Physiology and Biological Physics, University of Virginia School of Medicine, Charlottesville, VA, U.S.A.

**Keywords:** caveolae, caveolins, cell membranes, cryo-electron microscopy, membrane proteins, protein structure

## Abstract

The ability of cells to divide, migrate, relay signals, sense mechanical stimuli, and respond to stress all rely on nanoscale invaginations of the plasma membrane known as caveolae. The caveolins, a family of monotopic membrane proteins, form the inner layer of the caveolar coat. Caveolins have long been implicated in the generation of membrane curvature, in addition to serving as scaffolds for signaling proteins. Until recently, however, the molecular architecture of caveolins was unknown, making it impossible to understand how they operate at a mechanistic level. Over the past year, two independent lines of evidence — experimental and computational — have now converged to provide the first-ever glimpse into the structure of the oligomeric caveolin complexes that function as the building blocks of caveolae. Here, we summarize how these discoveries are transforming our understanding of this long-enigmatic protein family and their role in caveolae assembly and function. We present new models inspired by the structure for how caveolins oligomerize, remodel membranes, interact with their binding partners, and reorganize when mutated. Finally, we discuss emerging insights into structural differences among caveolin family members that enable them to support the proper functions of diverse tissues and organisms.

## Introduction

Nanoscale flask-shaped invaginations known as caveolae represent one of the most well-studied features of the plasma membrane. Found exclusively at the cell surface, caveolae exist in a wide range of tissues and cells and comprise up to 50% of the surface area of some cell types [1]. Caveolae have a stereotypical shape and size, with diameters on the order of 50–100 nm. While primarily static structures, they are metastable and can reversibly flatten and pinch off from the plasma membrane [2–6]. These features of caveolae endow them with the ability to regulate an enormous number of cellular events, including the ability of cells to divide, migrate, differentiate, sense and respond to mechanical stimuli and stress, protect and repair membrane damage, and communicate with other cells [7–12]. They also regulate numerous signaling pathways, function in lipid homeostasis and metabolism, and can be exploited by pathogens [13–17]. Studies of mice lacking key components of caveolae show they contribute to the proper function of many tissues and organs, including muscle, the cardiovascular system, and liver, as well as metabolic function [18–20]. Consistent with this, dysregulation of caveolae and their components has been linked to a staggering array of diseases in humans [12,21–26].

Much is known about the nuts and bolts of caveolae, including a ‘parts list' of their lipid and protein components as well as general mechanisms involved in their assembly, disassembly, and trafficking [2–6,10,11,23,27–30]. Despite this extensive body of knowledge, the structural features of caveolae required to assemble caveolae and support their many functions remain mysterious. Here, we summarize recent experimental and computational advances in our understanding of the structure of one of the core components of caveolae, caveolin-1 (CAV1). We also discuss the implications of these findings for how caveolins function as the building blocks of caveolae.

## The molecular architecture of the human CAV1 8S complex as revealed by cryo-electron microscopy (cryo-EM)

Caveolins are oligomeric integral membrane proteins that form the innermost layer of the caveolar coat [10,27]. They have a monotopic topology, allowing them to insert into the cytoplasmic leaflet of the plasma membrane with both their N- and C-termini facing the cytoplasm. Caveolins contain several functionally important domains including an oligomerization domain, scaffolding domain, intramembrane domain, and highly conserved signature motif [31]. However, until recently, our knowledge of the detailed structure of caveolins has been relatively limited [32,33].

**Figure 1.**
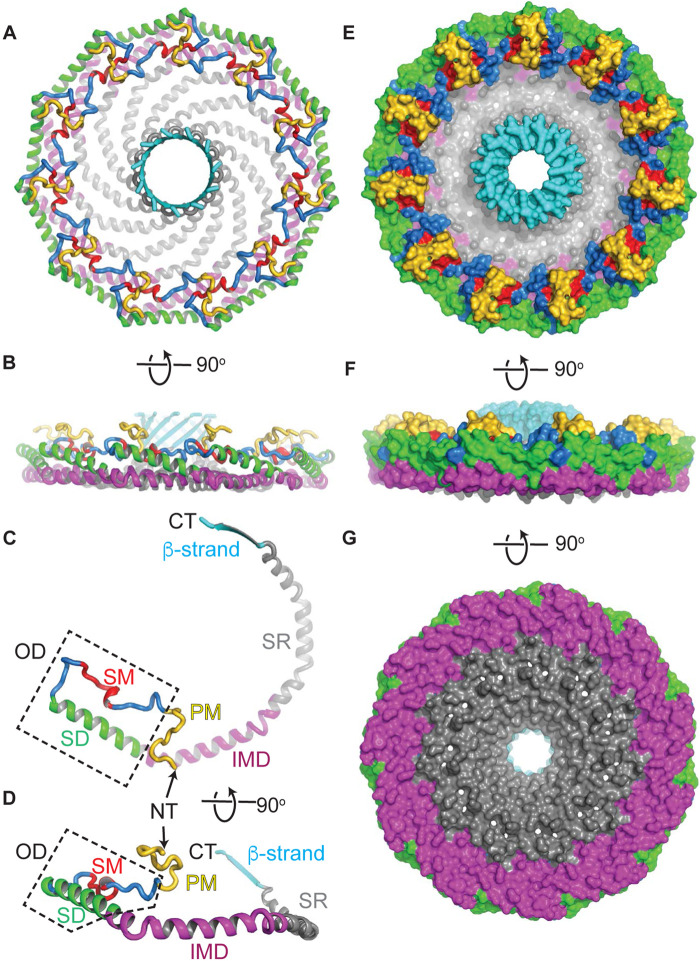
The cryo-EM structure of CAV1 reveals the overall architecture of the 8S complex, as well as the structure and organization of the CAV1 protomers. (**A** and **B**) Atomic model of CAV1 complex. (**A**) Cytoplasmic face of the complex. (**B**) Side view with membrane facing surface pointing down. (**C** and **D**) Structure of a CAV1 protomer, oriented to match panels **A** and **B**. (**E**–**G**) Space filling model of the CAV1 complex. (**E**) Cytoplasmic face of the complex. (**F**) Side view with membrane facing surface pointing down. (**G**) Membrane facing surface of the complex. Specific regions of each are color coded as follows: pin motif (PM), yellow; signature motif (SM), red; scaffolding domain (SD), green; intramembrane domain (IMD), purple; spoke-like region (SR), gray; β-strand, cyan. Note that the oligomerization domain includes the signature motif and scaffolding domain, as indicated by the boxed region in C and D. NT, N-terminus; CT, C-terminus. Adapted from Porta et al. [34] © The Authors, some rights reserved; exclusive licensee AAAS. Distributed under a Creative Commons Attribution NonCommercial License 4.0 (CC BY-NC) http://creativecommons.org/licenses/by-nc/4.0/.

A critical step forward in the field recently emerged with the determination of a 3.5 Å resolution cryo-EM structure of human CAV1 [34]. In the structure, CAV1 is organized into an oligomeric 8S complex, a state essential for caveolae biogenesis [34] (Figure 1). The complex is composed of 11 CAV1 protomers arranged symmetrically in a pinwheel-like arrangement around a central β-barrel. It is disc-like in shape, with a distinct outer rim, spoke region, and inner hub. The N and C-terminal domains are located on the cytoplasmic face of the disc. The opposing membrane-facing surface is both flat and hydrophobic, consistent with the expected monotopic topology of the complex. Amino acids 49–177 of the 178 residue CAV1 alpha isoform are resolved in the cryoEM structure. This is inclusive of the minimal region of CAV1 required to generate caveolae (residues 49–147) [35]. Notably absent from the cryoEM density map are the first 48 residues. This is in agreement with previous predictions that this region of the protein is unstructured [33,36].

The structure explains many previous observations about CAV1, such as why the oligomerization domain is required for the protein to form homo-oligomers and the functional importance of the signature motif. Furthermore, several new structural elements became apparent in the structure, including an N-terminal ‘pin motif' that stabilizes oligomers by forming a loop over the adjacent protomer, amphipathic helices that contribute to the spoke regions, and the parallel β-barrel in the center of the complex [34,37] (Figure 1).

Many features of CAV1 revealed by the structure were unexpected. One major surprise was that the protomers interact much more extensively than was previously anticipated. The resulting tight packing of adjacent protomers creates a structure that essentially lacks any empty space with the exception of the hydrophobic cavity of the central β-barrel. The disposition of the intramembrane domain was yet another surprise. In contrast with the prediction that the intramembrane domain forms a membrane-inserted hairpin [4,38–41], the structure reveals the intramembrane domain is part of a larger, flat membrane facing surface (Figure 1). The outward-facing surface of the outer rim of the complex is primarily hydrophobic. The rim is also raised with respect to the spoke region, which is a single helix deep. These unusual structural features of the complex have a number of intriguing implications, discussed further below.

## Alphafold2 correctly predicts many aspects of the structure of CAV1

The structure of CAV1 currently represents the first and only high resolution experimental structure available of any caveolin family member. It is thus important to understand to what extent computational approaches can be used to predict how the structure folds and behaves in a membrane environment. Prior to the determination of the cryo-EM structure, molecular dynamics simulations have been used to begin to address these questions [42–48]. However, these studies typically only considered monomeric caveolins or a truncated portion of the protein. It is unclear whether the results of these simulations reflect the properties of caveolin in the context of an intact 8S complex.

**Figure 2.**
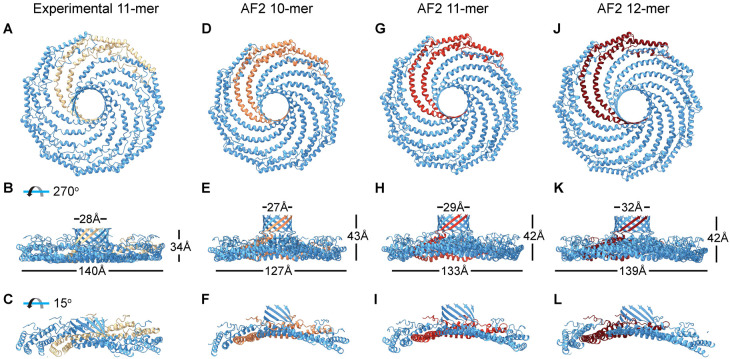
AlphaFold2 correctly assembles CAV1 protomers into oligomeric complexes and predicts multiple oligomeric states may exist. (**A**–**C**) Cryo-EM structure of CAV1 (PDB 7SC0). (**D**–**L**) Structures of CAV1 complexes predicted by AlphaFold2. Examples are shown for a 10-mer (**D**–**F**), 11-mer (**G**–**I**), and 12-mer (**J**–**L**). For each structure, two protomers are highlighted. Reprinted with permission from Gulsevin et al. [52]. Copyright (2022), used with permission from Elsevier.

Recent advances in protein structure prediction — in particular AlphaFold2 (AF2) — now make it possible to predict the structure of proteins and protein oligomers [49,50]. Caveolins are among the proteins contained in the AF2 database of proteins in the human proteome [51]. But, how good are these predictions? A recent side-by-side comparison reveals that the structure of the CAV1 monomer predicted by AF2 shares many of the features of a protomer extracted from the cryo-EM structure [52] (Figure 2). It also is capable of assembling 11 copies of CAV1 into oligomeric complexes that are organized and packed in a manner highly reminiscent of the CAV1 cryoEM structure (Figure 2). Strikingly, AF2 predicts CAV1 can assemble into closed discs containing between 7 and 15 protomers in addition to the experimentally observed 11-mer (Figure 2) [52]. This observation is quite interesting given that CAV1 oligomers have previously been proposed to contain between 7 and 16 copies of the protein [53–57]. Several of the complexes predicted by AF2 also show some evidence of curvature (Figure 2) [52]. This raises the possibility that the 8S complex exhibits some flexibility. Together, these findings suggest that AF2 can serve as a valuable tool to generate testable hypotheses regarding the structure and oligomerization of caveolins, as discussed further below.

## The structure of CAV1 sheds new light on several long-standing questions in the field

With the structure of CAV1 in hand, it is now possible to begin to address the many outstanding questions about how caveolins contribute to both the biogenesis and function of caveolae.

### How does CAV1 associate with membranes?

**Figure 3.**
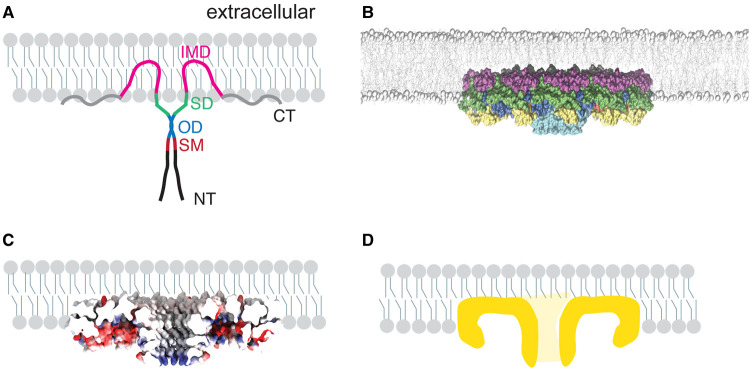
The cryo-EM structure of CAV1 suggests CAV1 associates with cell membranes in an unusual manner. (**A**) Classical model of the membrane topology and organization of CAV1. Signature motif (SM), red; scaffolding domain (SD), green; intramembrane domain (IMD), pink. Note that the oligomerization domain encompasses the signature motif and scaffolding domain in addition to the region in blue shown here. NT, N-terminus; CT, C-terminus. (**B**) Scale model of the CAV1 8S complex inserted into a membrane, as viewed from the side of the complex. The system was built with CHARMM-GUI [123–127] in all-atom representation. The membrane composition is the same on both leaflets and mimics the composition of the cytoplasmic leaflet of the red blood cell plasma membranes (containing 40 mol% cholesterol) [128]. The CAV1 complex was positioned so that the bottom of its number density overlapped with the bilayer center. The CHARMM-GUI software determined how many lipids to remove from the proximal leaflet in order to place the protein, based on known packing densities of the different types of lipids in the membrane. Lipid phosphate groups and cholesterol oxygens are shown as gray spheres, and lipid acyl chains are shown as gray sticks. Images were generated with the VMD software [129] and part of the bilayer was hidden to generate the side view of the complex. Regions of the CAV1 complex are color coded as follows: pin motif (PM), yellow; signature motif (SM), red; scaffolding domain (SD), green; intramembrane domain (IMD), purple; β-strand, cyan. Note that the spoke region is not visible in this representation. (**C**) Cut through of a space filling model of the cryoEM structure of the 8S complex embedded in a stylized membrane. The structure is color coded to show the position of charged residues. Blue, positive charge; red, negative charge; gray, neutral. (**D**) Simplified cartoon of the membrane topology of the CAV1 8S complex. Panel **A** is adapted from Porta et al. [34]. © The Authors, some rights reserved; exclusive licensee AAAS. Distributed under a Creative Commons Attribution NonCommercial License 4.0 (CC BY-NC) http://creativecommons.org/licenses/by-nc/4.0/.

It has long been known that CAV1 behaves as an integral membrane protein, but lacks a traditional transmembrane domain. Instead, it contains a long hydrophobic region termed the intramembrane domain [31,33,38]. Most previous models have suggested that CAV1 adopts a topology in which the intramembrane domain inserts as a hairpin into the cytosolic leaflet of the membrane, with the C-terminal domain lying at the membrane/cytosol interface (Figure 3A). Such models typically position the N-terminal region of the protein in the center of the oligomeric complex, reflecting a requirement for their oligomerization domain in supporting their self assembly (Figure 3A).

The finding that the 8S complex has a flat, hydrophobic membrane facing surface and a hydrophobic outer rim suggests the protein associates with membranes in a completely different manner than previously believed (Figure 3B–D). This new structure-based model predicts that the disc inserts deeply into the cytoplasmic leaflet of the membrane, displacing ∼250 lipids in the process (Figure 3B). In this scenario, the flat membrane facing surface is predicted to be solvated by a monolayer of lipids located on the extracellular leaflet of the membrane (Figure 3B,C). This unusual arrangement is likely to endow the protein with the ability to sort lipids [29]. For example, it is possible that specific lipids preferentially associate with the outer rim region and line its membrane-facing surface (Figure 3B,C). Lipids may also insert into the hydrophobic cavity of the β-barrel, which is capped by a charged residue at its C-terminal end (Figure 3C). This unique membrane topology undoubtably influences how CAV1 contributes to caveolae assembly, as well as its accessibility for binding to other proteins, as discussed further below. Furthermore, the presence of this large membrane-embedded disc also likely impacts the properties of the membrane in ways that have yet to be discovered

### How does CAV1 remodel membranes to drive caveolae assembly?

The most obvious function of caveolins is to sculpt membranes into the characteristic flask shape of caveolae. In non-muscle tissue, caveolae biogenesis requires the expression of both CAV1 and a second structural protein known as cavin-1 [4,30]. Current models suggest that caveolins form the inner membrane-associated coat of caveolae, while cavin-1, a peripheral membrane protein, packs on top of the caveolins [58,59]. Both CAV1 and cavin-1 are thought to contribute to caveolae assembly by bending membranes [4,30]. Very little is currently known about how CAV1 accomplishes this task. Based on the structure, several hypotheses can now be put forth.

One important question is to what extent CAV1 can bend membranes in the absence of cavin-1. This question has been addressed through ectopic expression of CAV1 in organisms that normally lack caveolins and caveolae [36,60–66]*.* In these systems, CAV1 expression can induce the formation of caveolae-like vesicles [36,60–67]. This has been particularly well studied in *E. coli,* where caveolin expression drives the assembly of structures termed heterologous (*h)*-caveolae [36,65,68]. *h*-caveolae formation is initiated by insertion of CAV1 oligomers into the inner membrane. This drives the formation of membrane invaginations that pinch off and ultimately accumulate in the *E. coli* cytoplasm in the form of intracellular vesicles highly enriched in caveolin [65]. Mutant forms of caveolin that are unable to oligomerize correctly are also unable to induce the formation of regularly shaped *h*-caveolae, suggesting these activities are linked. Intriguingly, *h*-caveolae are not round; instead, they exhibit polyhedral distortions [36]. How might caveolins generate these structures? A scale model based on the cryoEM CAV1 structure suggests that 8S complexes fit snugly on faces of polygons the size of *h*-caveolae, assuming dodecahedral symmetry [34]. This model raises the interesting possibility that at least in this simplified system, rather than to induce continuous membrane curvature the CAV1 complexes may function to stabilize the *flat* faces of *h*-caveolae, thus allowing the membrane to bend around the complex.

Unlike the simplified case of *h*-caveolae, generation of bona fide caveolae requires expression of cavin-1 as well as the cholesterol-dependent assembly of 8S complexes into higher order caveolin oligomers, detected biochemically in the form of 70S complexes [69]. Interestingly, mammalian caveolae have polygonal membrane profiles [59], and super resolution microscopy and cryo-electron tomography suggest that within caveolae, caveolin oligomers appear as polygonal units that organize into a polyhedral cage [57,58]. A structure-based model of packing of 8S complexes on dodecahedrons scaled to match the average size of mammalian caveolae indicates that more than one 8S complexes can be placed on each flat face [34], but it is not immediately obvious how they could arrange in a polyhedral cage. Missing from this picture is potential contributions of the disordered N-terminus of CAV1, which was not detected in the cryo-EM structure. It is possible that this region of CAV1 could potentially control the spacing of the 8S complexes and/or stabilize their interactions with each other to form the 70S complexes into a connected lattice. It is also currently unknown how rigid the 8S complexes are, or if they are flexible and capable of bending. In support of the latter possibility, AF2 predicts CAV1 complexes can exhibit some degree of curvature [52] (Figure 2). The extent to which cavin-1, rather than CAV1, could potentially also play a role in defining the average size of caveolae is also not yet clear. Thus, more work is needed to resolve exactly how caveolins and cavins pack together and cooperate with lipids to build mammalian caveolae.

### What sites on CAV1 are available for binding to other proteins?

One of the most longstanding hypotheses in the field is that caveolae serve a scaffolding function [70,71]. The scaffolding hypothesis postulates that proteins containing a consensus caveolin binding motif interact with the scaffolding domain of caveolins [72]. However, bioinformatic and structural evidence have called several of the underlying assumptions of the model into question [73,74]. For example, caveolin binding motifs do not assume a common structural motif. They also are often buried in the hydrophobic core of proteins that contain them, suggesting they are unlikely to be readily accessible for binding to other proteins [73,74]. A recent cell-free screen for caveolin interacting proteins using a form of membrane-inserted caveolin also failed to detect several interactions of several of caveolin's proposed binding partners [66]. This raises the question of whether the scaffolding domain of caveolin itself is accessible for binding. Based on the cryo-EM structure, the scaffolding domain is located on the outer rim of the complex, facing both the cytoplasm and membrane. It thus could potentially be available to participate in protein–protein interactions (Figures 1 and 3). How much of the scaffolding domain is accessible from the cytoplasm likely depends on how deeply the entire complex is inserted into the membrane, as well as the position of the disordered N-terminus, which is not detected in the structure.

Independent of the scaffolding domain, several surfaces of the complex, including the spoke region and β-barrel, are predicted to be accessible from the cytoplasm. They could thus potentially serve as protein–protein interactions interfaces (Figures 1 and 3). The disordered N-terminus has recently been implicated in interactions with cavin-1 [75], and several other protein–protein interactions have also been mapped to this region of CAV1 [76,77]. The N-terminus also contains a tyrosine phosphorylation site, and binding of several interacting partners have been reported to depend on the phosphorylation state of the protein [66]. More work will be needed to fully explore the potential functional importance of these regions as binding sites both inside and outside of caveolae.

### What structural features distinguish different caveolin family members, and how does this regulate their specific functions?

**Figure 4.**
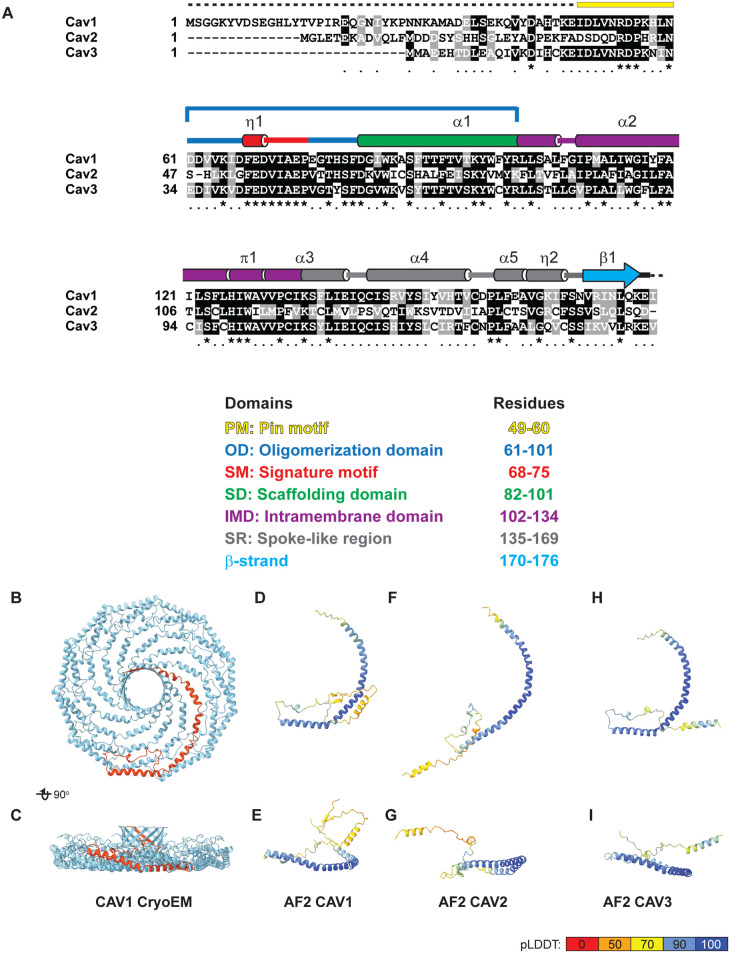
Sequence alignment and AF2 predictions of the structure of human CAV1, CAV2, and CAV3 monomers highlight their similarities and differences. (**A**) Sequence alignment showing the position of key domains and secondary structure elements identified in the CAV1 structure. (**B** and **C**) CryoEM structure of CAV1 8S complex (PDB 7SC0). A single protomer is highlighted in pink. (**D**–**I**) AF2 predicts CAV1, CAV2, and CAV3 monomers are all primarily helical, but have somewhat different tertiary structures. Predicted structures of CAV1 (**D** and **E**), CAV2 (**F** and **G**) and CAV3 (**H** and **I**) monomers are all taken from the AF2 database [51]. Confidence levels of the predictions in **C**–**H** are rendered on the models using pLDDT (predicted local-distance difference test) values [49]. Note that the AF2 predictions include the full range of residues for each protein, including regions of the N-termini that are not predicted with high certainty. Panels **B**–**I** are adapted from Han et al. [89]. Distributed under a Creative Commons Attribution NonCommercial License 4.0 (CC BY-NC) https://creativecommons.org/licenses/by-nc/4.0/.

Three caveolin family members exist in humans [31]. CAV1- the founding member of the gene family - is essential for caveolae biogenesis in most tissues. Caveolin-2 (CAV2) is typically co-expressed with CAV1, whereas caveolin-3 (CAV3) is expressed exclusively in muscle. The three caveolin family members share a highly conserved motif known as the signature motif (Figure 4), but otherwise differ in sequence similarity and function. CAV1 and CAV3 form oligomeric complexes and are capable of assembling caveolae, but their interactomes differ [78,79]. In contrast, CAV2 is unable to form 8S complexes and cannot assemble caveolae on its own [62,69,80,81]. How the structural feature of the caveolin family members differ from one another, and how their unique structural elements and functions are connected remain unknown [19,82–85]. It is now possible to begin to address these important questions.

Consistent with their shared ability to support caveolae biogenesis, the overall size and shape of CAV1 and CAV3 complexes are generally similar as reported by negative stain single particle electron microscopy [59,68,86]. Furthermore, AF2 predicts many of the structural features of CAV3 monomers closely resemble those of CAV1 (Figure 4). One notable difference is that CAV3 is predicted by AF2 to contain an alpha helix in its N-terminal domain with a relatively high degree of confidence (Figure 4), whereas the N-terminal most residues of CAV1 are most likely disordered based on their absence from the cryoEM structure. This N-terminal helix was recently proposed to regulate trafficking of CAV3 out of the ER, ultimately contributing to different proteostatic fates of CAV1 and CAV3 [87]. Another notable difference is that CAV3 contains 6 cysteines that serve as potential palmitoylation sites per protomer, compared with the three for the case of CAV1 [88]. One of these sites on CAV3 can undergo glutathiolation, and this modification disrupts the interaction of CAV3 with heterotrimeric G protein alpha subunits [88]. Interestingly, CAV2 is predicted by AF2 to share many structural elements with CAV1 and CAV3 (Figure 4) [89]. An important distinction is that the CAV2 monomer is not predicted to bend between helix 1 and helix 2. Whether the absence of this structural element prevents CAV2 from self-assembling into 8S complexes, as well as how the protein is able to hetero-oligomerize with CAV1 despite their structural differences remains to be determined.

### What are the structural consequences of disease-associated mutations in CAV1?

Many of the pathophysiological effects of CAV1 are thought to be linked to changes in expression level. There are however several examples of disease-associated mutations of CAV1, and even more for muscle-specific CAV3 [24,90–92]. This raises the important question of how mutations in caveolins disrupt caveolae assembly and function.

**Figure 5.**
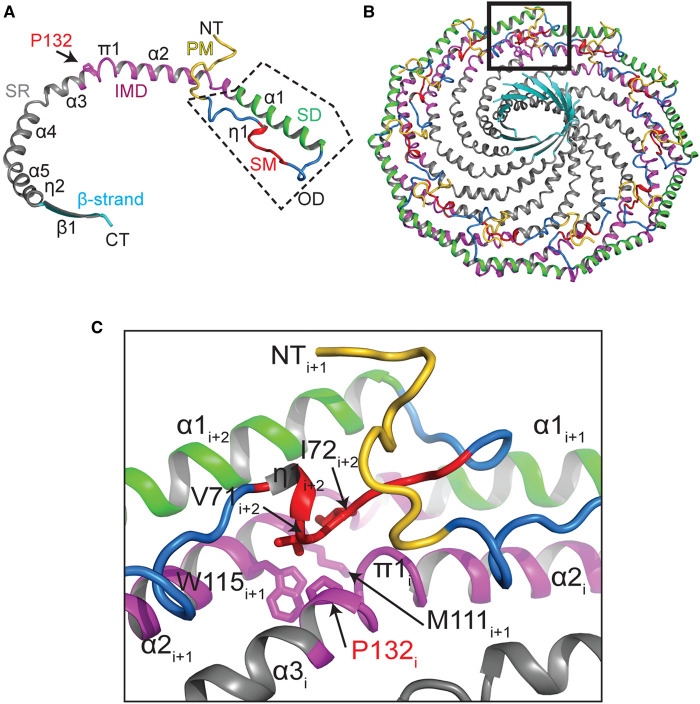
P132 is positioned at a critical protomer–protomer interface in the CAV1 8S complex. (**A**) Position of P132 highlighted on a CAV1 protomer. (**B**) View of the entire CAV1 8S complex with the position of P132 highlighted in the boxed region. (**C**) Zoom of boxed region from panel **B**. Specific regions of each are color coded as follows: pin motif (PM), yellow; signature motif (SM), red; scaffolding domain (SD), green; intramembrane domain (IMD), purple; spoke-like region (SR), gray; β-strand, cyan. Note that the oligomerization domain includes the signature motif and scaffolding domain, as indicated by the boxed region in **C** and **D**. NT, N-terminus; CT, C-terminus. Adapted from Han et al. [102]. © 2023 THE AUTHORS. Published by Elsevier Inc on behalf of American Society for Biochemistry and Molecular Biology. Distributed under a Creative Commons Attribution 4.0 International (CC BY 4.0). License https://creativecommons.org/licenses/by/4.0/.

Some disease mutations interfere with the ability of 8S complexes to assemble correctly, in turn preventing caveolae biogenesis. The most famous example is P132L, a mutation in one of the most highly conserved residues in CAV1 linked to breast and lung cancer [93–97]. Mutations in an equivalent residue in CAV3, P105L (sometimes referred to as P104L) are associated with muscular dystrophy [24,98–100]. These mutations are known to interfere with the ability of caveolins to oligomerize correctly [93,99]. Previous studies suggested that P132L may extend a helical region of CAV1 [101], but why this would impact oligomerization was not entirely clear. Based on experimental and predicted structures, it is now apparent that P132 and P105L are positioned at a critical protomer–protomer interface, helping to explain the source of their oligomerization defects [87,102] (Figure 5).

**Figure 6.**
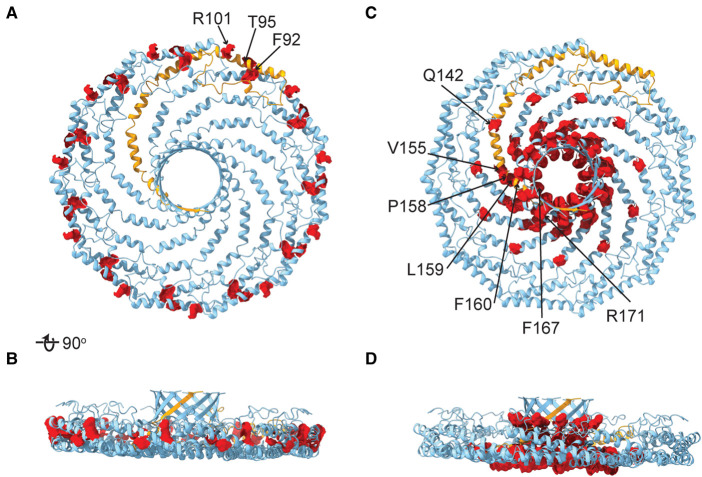
Residues in CAV1 that undergo disease-associated mutations are clustered in the scaffolding domain and spoke region/β-barrel. (**A** and **B**) Positions of residues on the scaffolding domain superimposed on the cryoEM structure of CAV1 (PDB 7SC0). F92, a residue in the scaffolding domain that disrupts caveolin-dependent signaling but preserves caveolae formation when mutated to alanine [130] is shown for comparison. (**C** and **D**) Position of residues located in the spoke region and β-barrel. In each panel, a single protomer is highlighted in orange and residues that undergo disease-associated mutations are highlighted in red.

Mutations in CAV1 are also linked to pulmonary arterial hypertension and lipodystrophies [20,26,103–115]. Two of the best-studied examples include heterozygous frameshift mutations in the C-terminal region of the protein, P158P and F160X [110–114] (Figure 6). These mutations appear to interfere with caveolae via a different mechanism than for the case of the P132L mutation. The F160X frameshift leads to a premature truncation of the protein [105,112,113]. This mutation decreases the stability of 8S complexes, but the mutant complexes are still capable of supporting caveolae formation [105,112]. The P158P frameshift on the other hand gives rise to a novel C-terminus [111]. This introduces a *de novo* ER exit signal that interferes with the delivery of the mutant protein to the cell surface when expressed individually [110,114]. It is nevertheless still capable of forming 8S complexes either on its own or together with WT CAV1 [110]. Both of these mutant proteins are predicted to interfere with the formation of the central β-barrel. Negative stain electron microscopy of purified complexes formed by either F160X or P158P mutant proteins confirms that they form oligomeric complexes, but the complexes are considerably less regular than those formed by WT CAV1 [68]. Both mutant proteins also lack a central protrusion, consistent with the absence of the β-barrel [68]. The mechanisms by which the β-barrel contributes to the formation of fully functional caveolae, as well as how its absence gives rise to disease require further investigation.

Strikingly, several other disease-associated mutations in CAV1 identified in patients with pulmonary arterial hypertension and/or lipodystrophy syndromes [34,93,101,105,107,111–113,115] cluster near the residues P158 and F160 in the structure, whereas another set of mutations are located in the scaffolding domain (Figure 6). It is tempting to hypothesize that the mutations that cluster in the scaffolding region interfere with oligomerization, whereas those found closer to the center of the complex disrupt the central β-barrel. These possibilities remain to be experimentally tested.

### What functions do caveolins fulfill outside of vertebrates?

How the properties and functions of caveolins vary across evolutionary space is still largely unexplored [35,89]. Caveolins were previously thought to exist only in metazoans [35,116,117]. However, more recent evidence suggests caveolin family members are also found in a subset of choanoflagellates, the closest living relatives of metazoans [89,118]. On the other hand, expression of cavins, which are also required for caveolae assembly, is limited to vertebrates [119]. This implies that caveolins function outside of caveolae in many organisms and raises the important question of what these functions are and how caveolin structure is optimized to accommodate them. AF2 predicts that many evolutionarily diverse caveolins share the ability to form oligomeric complexes, and that association with membranes is one of the most well-conserved features of the proteins, although this remains to be validated experimentally [89]. There is growing evidence that caveolins can generate functionally relevant membrane curvature in cells that lack cavins, for example in invertebrate chordate Ciona spp. [120]. Very recent work suggests that caveolins may function as membrane buffers outside of caveolae by forming a newly described class of curved membrane structures known as dolines [121,122]. More work will be required to elucidate the full range of organisms where these structures are operative and to dissect the structural features of caveolins that enable them to function both inside and outside of caveolae.

## Conclusion

In summary, the newly determined structure of CAV1 is transforming what we thought we knew about this essential building block of caveolae. It challenges long-held models for how caveolins oligomerize, associate with membranes, and interact with their binding partners. It also provides a much-needed framework to probe the structural consequences of disease-associated mutations. Parallel advances in protein structure prediction are extending these discoveries even further by uncovering similarities and differences among caveolin family members in humans and across diverse organisms. Mechanistic insights into how caveolins and caveolae function are sure to follow.

## Perspectives

- Caveolins and caveolae fulfill wide-ranging roles in cell and organismal physiology, and defects in caveolins and caveolae give rise to multiple diseases including cancer, cardiovascular disease, and lipodystrophies in humans. The membrane protein caveolin-1, an essential building block of caveolae, serves as an archetype for understanding how the structure and function of caveolins are linked.
- Cryo-electron microscopy has now revealed a high-resolution structure of human caveolin-1 in a functional form that is essential for caveolae biogenesis. In parallel, recent computational advances are now making it possible to predict the structure of caveolins with reasonable accuracy. The insights emerging from these structures are re-defining what we thought we understood about caveolins.
- The structure of caveolin is inspiring fresh hypotheses about how caveolins assemble into functional complexes, bind to other proteins and lipids, insert into and bend biological membranes, contribute to disease progression, and support cell- and tissue-specific functions. Much exciting work lies ahead to test these new ideas.

## Abbreviations

AF2: AlphaFold2
CAV1: caveolin-1
CAV2: caveolin-2
CAV3: caveolin-3
CT: C-terminus
IMD: intramembrane domain
NT: N-terminus
PM: pin motif
SD: scaffolding domain
SM: signature motif
SR: spoke-like region
